# An Improved Multi-temporal and Multi-feature Tea Plantation Identification Method Using Sentinel-2 Imagery

**DOI:** 10.3390/s19092087

**Published:** 2019-05-05

**Authors:** Jun Zhu, Ziwu Pan, Hang Wang, Peijie Huang, Jiulin Sun, Fen Qin, Zhenzhen Liu

**Affiliations:** 1 College of Environment and Planning, Henan University, Kaifeng 475004, China; zhujun@vip.henu.edu.cn (J.Z.); wugemicheal@163.com (Z.P.); wanghang20001@163.com (H.W.); liuzhenzhengis@foxmail.com (Z.L.); 2 Laboratory of Geospatial Technology for the Middle and Lower Yellow River Regions, Ministry of Education, Henan University, Kaifeng 475004, China; 3 Department of Geography, Hanshan Normal University, Chaozhou 521041, China; 4 Yellow River Engineering Consulting Co., Ltd, Zhengzhou 450003, China; pjhuang517@126.com; 5 Institute of Geographic Sciences and Natural Resources Research, Beijing 100101, China; sunjl@igsnrr.ac.cn; 6 Henan Industrial Technology Academy of Spatio-Temporal Big Data, Henan University, Kaifeng 475004, China

**Keywords:** remote sensing, Sentinel-2, tea plantation identification, Random Forest algorithm, feature selection, China

## Abstract

As tea is an important economic crop in many regions, efficient and accurate methods for remotely identifying tea plantations are essential for the implementation of sustainable tea practices and for periodic monitoring. In this study, we developed and tested a method for tea plantation identification based on multi-temporal Sentinel-2 images and a multi-feature Random Forest (RF) algorithm. We used phenological patterns of tea cultivation in China’s Shihe District (such as the multiple annual growing, harvest, and pruning stages) to extracted multi-temporal Sentinel-2 MSI bands, their derived first spectral derivative, NDVI and textures, and topographic features. We then assessed feature importance using RF analysis; the optimal combination of features was used as the input variable for RF classification to extract tea plantations in the study area. A comparison of our results with those achieved using the Support Vector Machine method and statistical data from local government departments showed that our method had a higher producer’s accuracy (96.57%) and user’s accuracy (96.02%). These results demonstrate that: (1) multi-temporal and multi-feature classification can improve the accuracy of tea plantation recognition, (2) RF classification feature importance analysis can effectively reduce feature dimensions and improve classification efficiency, and (3) the combination of multi-temporal Sentinel-2 images and the RF algorithm improves our ability to identify and monitor tea plantations.

## 1. Introduction

Tea is an economically significant crop in global agriculture [1,2] and an important economic engine in many developing countries [3]. The global tea industry has developed rapidly since the beginning of this century; according to the International Tea Commission, the global tea plantation area in 2015 was 4.52 million ha, a 70.6% increase over the 2.65 million ha in 2000 (http://www.inttea.com/). Tea production played an important role in the development of the Chinese agricultural economy [4], and China is now the world’s largest tea producer, with 2.79 million ha under cultivation in 2015, accounting for 61.7% of global tea plantation area [5,6]. Determining the spatial distribution and area of tea plantations in a timely and accurate manner is of great significance for analysing and regulating the industry, optimizing the regional distribution of tea production, and promoting its sustainable development [7]. This can also provide basic data for yield estimation, disease analysis, environmental effects, and other research interests.

It is expensive and time-consuming to monitor the large-scale spatial distribution and area of tea plantations using traditional surveys and statistics. However, remote sensing technology enables efficient spatiotemporal observation of land-use/land-cover (LULC) change processes [8,9]. The existing research on tea plantation recognition has used a variety of data sources and classification methods. Ghosh et al. [10] classified tea plantations and other land cover types in Assam, India, by visual interpretation of IRS LISS II images, but the classification accuracy was relatively low due to inherent uncertainty of this approach as well as limitations of the satellite data. A subsequent study used decision tree classifiers with these images, achieving an overall classification accuracy of 88% [11]. He et al. [12] used an artificial neural network (ANN) ensemble and maximum-likelihood classification (MLC) to classify land cover based on Landsat TM and topographical data, achieving 81.03% overall accuracy and 43.33% for tea and mulberry plantations. 

As remote sensing technology continues to develop, many high-resolution images have been used for tea plantation extraction. Fauziana et al. [13] utilized SPOT-7 imagery to determine the tea/non-tea fractions using linear spectral mixture analysis; this produced high-accuracy results but overestimated certain tea areas. Dihkan et al. [9] extracted spectral and textural features from high-resolution digital aerial images and used a modified vegetation index with a Support Vector Machine (SVM) algorithm to classify LULC types with producer’s and user’s accuracies of 92.09% and 94.68%, respectively. Xu et al. [4] used the decision tree method with spectral features, normalized difference vegetation index (NDVI), and direction intensity features from ZY-3 imagery to extract the tea plantation data; producer’s and user’s accuracies in a mountainous area were 90.05% and 85.58%, respectively, while those in a plains area were 71.84% and 73.99%. Xu [14] identified tea plantations in GF-1 imagery using the multi-level rule classification method based on object-oriented and multi-source data fusion, obtaining producer’s and user’s accuracies of 88.2% and 87.7%, respectively. Chuang and Shiu [15] used the spectral and texture features of WorldView-2 imagery to interpret tea-related LULC; by comparing four classification methods, they found that MLC achieved the highest producer’s and user’s accuracies (87.31% and 95.51%, respectively). Yang [16] also used WorldView-2 imagery to classify vegetation based on spectral features, NDVI, and fingerprint texture features, with producer’s and user’s accuracies of 83.3% and 93.67%, respectively.

As tea is a perennial woody evergreen plant, its spectral characteristics are easily confused with similar vegetation such as orchards and bushes, so it is difficult to achieve ideal accuracy for tea plantation identification using only spectral features [9]. Previous studies have mainly used single-temporal medium- or high-resolution optical images for tea plantation recognition. The identification accuracy when using the former was low, though much improved when using the latter. However, high-resolution image data have generally been expensive and unable to provide high temporal resolutions, making it difficult to identify and monitor tea plantations at high temporal frequencies over large areas. In this context, Sentinel-2 imagery is among the best options for regular monitoring of tea plantations due to its high spectral and spatial resolution, continuity, affordability and access, and its history of successful application to multi-temporal classification research for crops and other vegetation. In this study, we developed an improved method for identifying tea plantations based on multiple features (spectral reflectance, first-derivative spectra, NDVI, and textures) derived from multi-temporal Sentinel-2 images and topographic features, verified the accuracy of our method, and produced a spatially explicit tea plantation map in the chosen study area. 

## 2. Materials and Methods 

### 2.1. Study Area 

**Figure 1:**
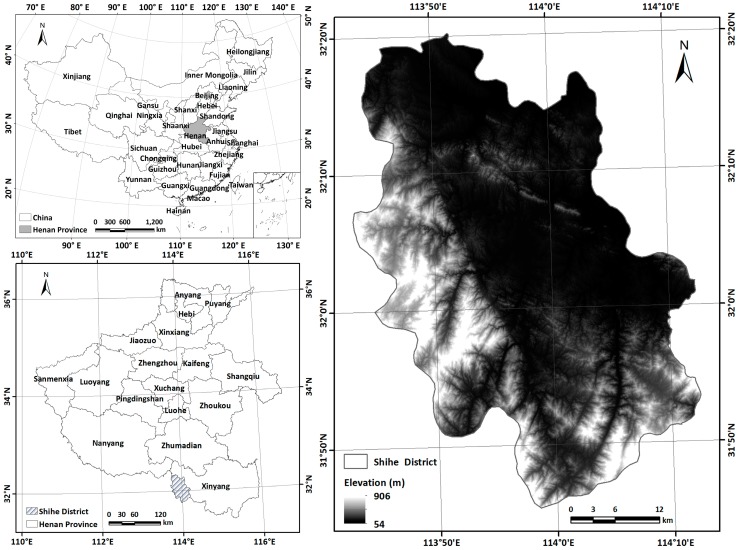
Study area location in Henan Province, China, and digital elevation model (DEM).

We selected the Shihe District (Figure 1) in the western part of Xinyang City, Henan Province, China as our study area. This is a typical region in the Jiangbei Tea District, one of the four largest tea districts in China, and covers 1783 km^2^ between 113°42′36″ E to 114°08′34˝ E and 31°24′06″ N to 32°33′00″. The area has a continental monsoon climate within the transition from subtropical to warm temperate zones, with an average temperature of 15.1 °C and an annual precipitation of 1109.11 mm. The elevation ranges from 54 to 906 m, with the highest terrain in the Tongbai and Dabie Mountains to the southwest and the lowest in the northeastern plains along the Huaihe River. This district is the largest county for green tea production in China and is the origin and main production area of the famous Chinese teas “Xinyang Maojian” and “Xinyang Red”. In 2004, it was named the "Hometown of Chinese Tea" by the state forestry administration of the People’s Republic of China. At present, tea plantations in the region cover 472.56 km^2^ [17], accounting for 26.5% of the district’s total area.

**Figure 2:**
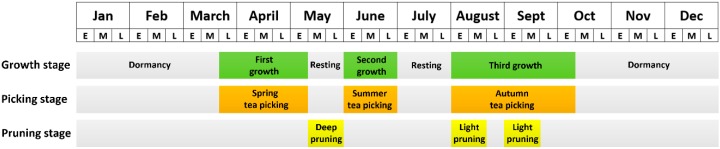
Annual growth, picking, and pruning stages of tea in the study area delineated by the first ten (E), middle ten (M), and last ten (L) days of each month.

**Figure 3:**
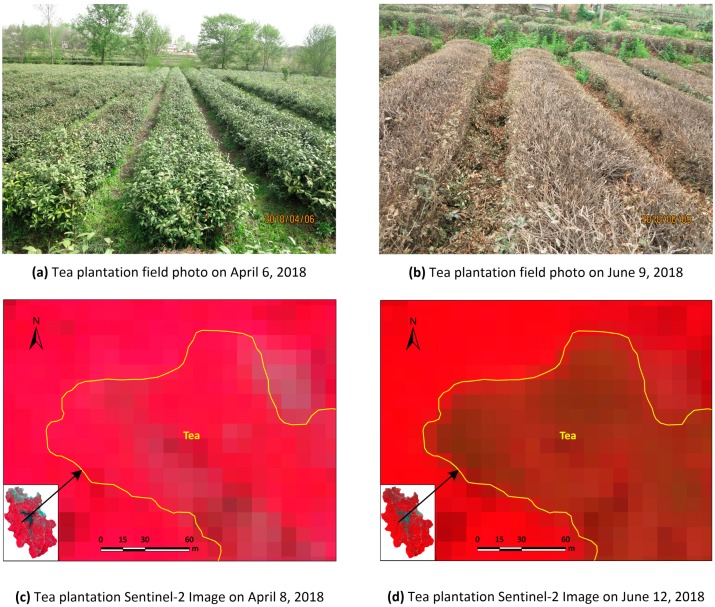
Effect of pruning on tea plantations in the study area: (**a**,**b**) field photos of tea plantations before and after pruning, respectively; (**c**,**d**) Sentinel-2 false colour images of tea plantations before and after pruning, respectively.

Tea in China follows a regular annual growth cycle that begins in late March and goes through three growth stages and two rest stages before returning to dormancy in mid-late October [18]. Farmers usually pick tea during the growing stages; in the study area, spring tea begins to be picked in late March or early April through mid-late May, summer tea is picked in early June through early July, and autumn tea is picked in early August through early October. Tea plants in the study area are pruned three times a year to regulate and control their branching habits, promote hierarchy and health, and prevent pests and diseases; proper pruning can prolong the life of stable/high-yielding, high-quality tea plants [19,20]. A deep pruning is usually conducted in May following the spring harvest and two light prunings are conducted in August and September (Figure 2). Our field investigations showed that there was a significant difference in appearance between tea plantations before and after deep pruning (Figure 3).

### 2.2. Data and Preprocessing

#### 2.2.1. Sentinel-2 Image Data

The Sentinel-2 satellite images (Level-1C S2) were downloaded from the European Space Agency’s (ESA) Sentinel Scientific Data Hub. We selected images from four different seasons (18 April 2018; 12 June 2018; 15 September 2017; and 19 December 2017) to account for changes in tea growth influenced by picking, pruning, weather conditions, and image availability. We choose the blue (B2), green (B3), red (B4), and near-infrared (B8) bands with 10 m resolution and four red-edge (B5, B6, B7, and B8A) bands with 20 m resolution. Radiation calibration and atmospheric correction of the images, as well as resampling of the red-edge bands from 20 m to 10 m, were carried out in ENVI 5.3 and ENVI 5.5.

#### 2.2.2. Digital Elevation Model (DEM) Data

We obtained 30 m DEM data from NASA Shuttle Radar Topography Mission (SRTM), and used them, as well as slope and aspect data derived therefrom, as terrain feature variables for tea plantation identification and mapping.

#### 2.2.3. Ground Survey Data and Sample Datasets

**Figure 4:**
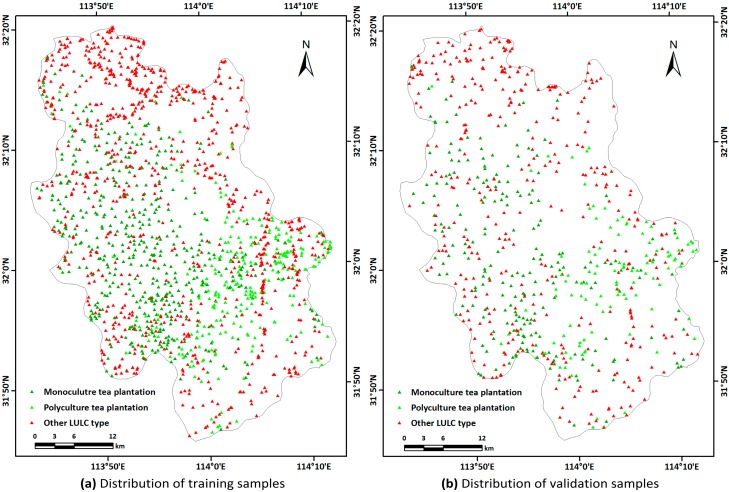
Distribution of samples in the study area: (**a**) 20,565 training sample pixels, of which 5654 are tea plantations; (**b**) 8756 validation sample pixels, of which 2449 are tea plantations.

The sample bank of the study area was established using ground survey data and Google Earth high-resolution remote sensing image data. We used ground surveys in April and June, 2018, to collect 410 samples of typical land-use types, including tea plantations, forest, cropland, built-up, and water. 2259 polygonal samples were obtained by two researchers’ independent visual interpretation of Google Earth imagery: the number of actual pixels was 29,321, of which 7828 were tea plantations and 21,493 were other categories. Thus, tea plantation samples accounted for 27% of the total, consistent with the actual proportion of tea plantation area in the study area. Stratified random sampling of the samples was carried out in ArcGIS 10.2, 70% of which were training samples with the rest serving as validation samples (Figure 4).

### 2.3. Methods

**Figure 5:**
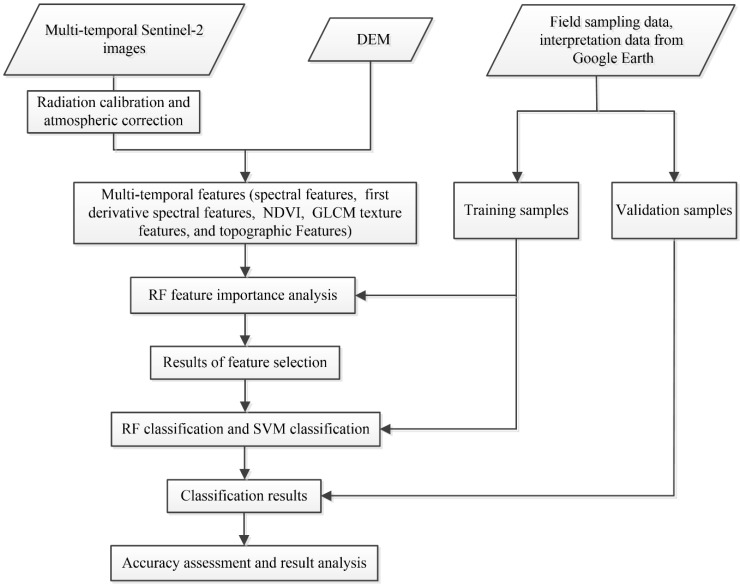
Flowchart for the tea plantation identification method proposed in this study.

Based on the unique characteristics of tea plantations, we developed a method based on the multi-temporal and multi-feature Sentinel-2 images to distinguish tea plantations from their surrounding areas (Figure 5).

#### 2.3.1. Feature Analysis and Selection

**Figure 6:**
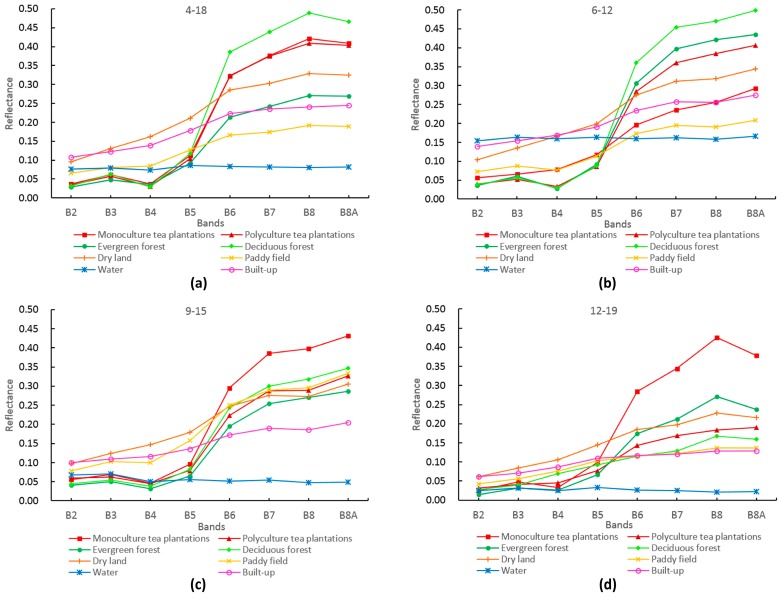
Spectral curves of the eight LULC types in the study area on (**a**) 18 April 2018, (**b**) 12 June 2018, (**c**) 15 September 2017, and (**d**) 19 December 2017.

The main LULC types in the study area were tea plantations, evergreen forests, deciduous forests, dry land, paddy fields, built-up, and water. Our field investigations showed that many southeastern tea areas were interplanted with agroforestry species (such as chestnuts), so tea plantations were subdivided into two types: monoculture and polyculture. We extracted the spectral reflectance of different LULC types from Sentinel-2 multi-temporal images using the sample data, then calculated the mean reflectance of each type and analysed the spectral differences between tea plantations and the rest (Figure 6). In the blue (B2), green (B3), red (B4), and red-edge (B5) bands, the spectral characteristics of both tea plantation types were similar to those of evergreen forest, deciduous forest, paddy fields, and dry land. In the near-infrared (B8) and red-edge (B6, B7, B8A) bands, although the reflectance of water was obviously distinct, there were different degrees of confusion between the two tea plantation types and others in different seasons. Therefore, it was difficult to clearly identify tea plantations using only the spectral features of the 8 bands, making it necessary to use auxiliary information such as spectral derivatives, NDVI, textures, and topographical features to improve the identification accuracy.

**Figure 7:**
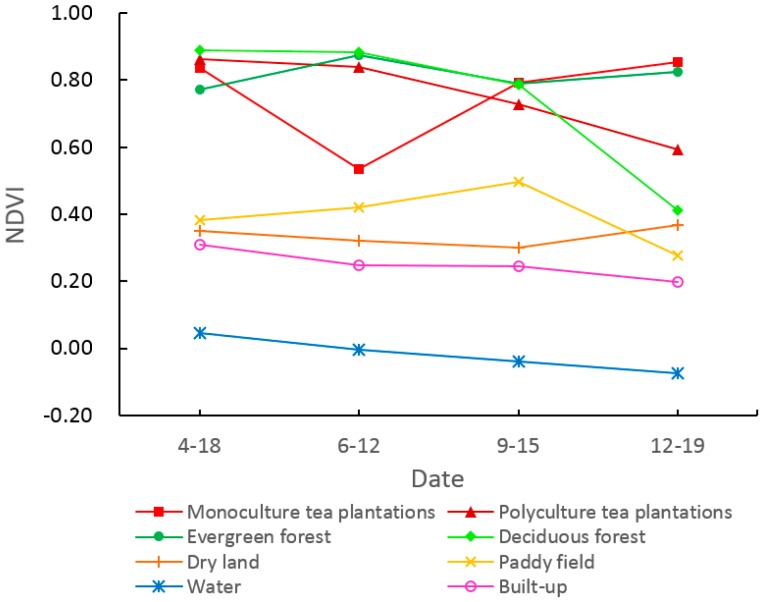
NDVI values for the eight LULC types on the four image dates.

When the NDVI was plotted for the eight typical LULC types on each of the four imagery dates (Figure 7), three clear observations could be made. First, the NDVI of paddy fields, dry land, built-up, and water was obviously different from tea plantations and forests. Second, the NDVI of monoculture tea plantations was similar to polyculture tea plantations, evergreen forest, and deciduous forest in April and September but was significantly lower in June. This was because the vegetative characteristics of monoculture tea plantations were missing in early June (after harvest and extensive pruning) but this effect was buffered by the foliage of interplanted (chestnut) trees in the polyculture tea plantations. Third, the NDVI of monoculture tea plantations and evergreen forest was very similar in December while that of polyculture tea plantations and deciduous forests was lower. This was because the tea plants were dormant in December but retained their leaves, such that the NDVI of monoculture tea plantations was similar to evergreen forest, while that of deciduous forests (following leaf drop) was lowest, and that of polyculture tea plantations reflected the combination of evergreen tea plants and deciduous interplanted trees (like chestnuts). In April, June and September, chestnut trees were in the germination and leaf development stage, rapid growth stage, and fruit ripening stage, respectively, so their NDVI remained high, while by December, chestnut trees had dropped their leaves, pulling the NDVI of polyculture tea plantations downward. According to the field survey, most forest in the study area was deciduous, so the difference in NDVI between December and June can be used to distinguishing monoculture tea plantations, polyculture tea plantations, and most forest areas. December was a good period in which to distinguish polyculture tea from other similar types.

Solving the first derivative of spectral reflectance can reflect the change rate of the original spectral curve and enhance the slight differences in slope for vegetation, better reflecting the essential characteristics in different growth stages and increasing the separability of land cover types [21,22]. Texture can also reflect the spatial structure characteristics of objects [23]. Compared with other LULC types, the spatial textural features of tea plantations were more significant. Adding these features to the tea plantation extraction process can thus make up for the lack of spatial information for spectral features and improve the classification accuracy [24]. In the study area, tea plantations were mostly distributed in low mountainous and hilly areas, such that topographic conditions including elevation, slope, and aspect directly affected the strip characteristics of tea plantations established along contour lines. Therefore, we extracted a total of 325 spectral, NDVI, and GLCM textural features from the four Sentinel-2 images, and topographic features as input variables (Table 1).

#### 2.3.2. Classification Method

Random Forest (RF) is an ensemble learning algorithm proposed by Breiman that consists of multiple decision trees or classified regression trees [25]. Each tree is constructed by a certain number of random samples and random feature training [26,27,28]. The basic algorithm flow of RF classification is as follows: (1) Using the bootstrapping sampling method, two-thirds of the data are extracted as training samples (called in-bag data) and the remaining one-third are validation samples (called out-of-bag (OOB) data). The latter can be used to estimate the internal error. (2) A classification and regression tree is constructed for each training sample set to generate a random forest consisting of N trees. In the growth process of each tree, *m* is randomly selected from all the features *M* (usually *m* = $\sqrt{M}$). In *m* features, the optimal segmentation feature is selected according to the Gini coefficient, calculated as follows:

(1) $$Gini=1-\sum_{C}p^{2}\left(C/N\right)$$

where *C* is the number of classes, *N* is the number of trees, and *P* represents the probability of belonging to *C*. (3) Combining the classification results of *N* decision trees, the final classification results are determined by the majority voting principle.

Multi-temporal and multi-category features are helpful for improving the recognition accuracy of LULC types, but the large dimensions of features involved in classification will lead to increasing computational complexity and decreasing computational efficiency of the classifier, and not every feature will have a significant impact on the classification accuracy. Therefore, it is necessary to extract the importance information of features and obtain a feature subset as small as possible by eliminating redundant or irrelevant features without significantly reducing the classification accuracy. The RF algorithm calculates variable importance using OOB data errors. First, for each tree *i* in the random forest, errOOB1^f^_i_ is calculated by using the OOB data of feature *f*; then noise interference is added to feature *f* of OOB^f^_i_ data randomly, and errOOB2 ^f^_i_ is calculated again; the formula for calculating feature *f* importance is as follows:

(2) $$FI^{f}=\frac{1}{N}\sum_{N}\left(\mathrm{errOOB}2_{i}^{f}-\mathrm{errOOB}1_{i}^{f}\right)$$

When random noise is added, if the classification accuracy of the OOB data decreases dramatically (that is, errOOB2 increases), this shows that this feature has a clear impact on the prediction results of samples; in other words, it is of high importance.

#### 2.3.3. Determination of Random Forest Parameter

**Figure 8:**
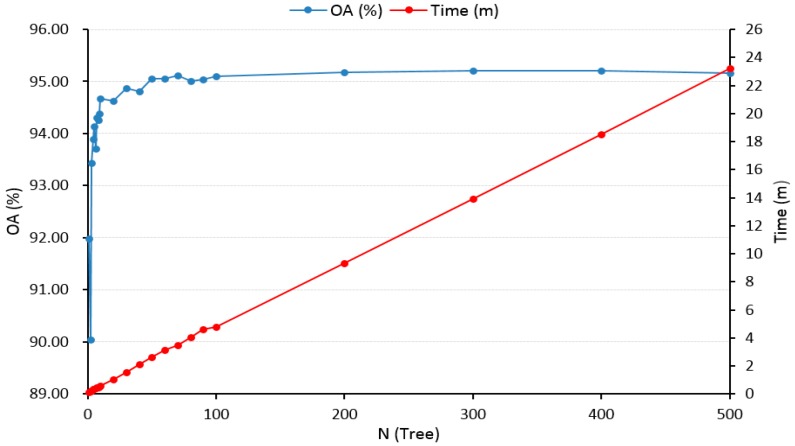
Effect of decision tree number on overall classification accuracy and modeling time in the RF model used in this study.

We built the RF classification model using EnMap-Box 2.2 software [29,30], initially selecting 1–500 decision trees for parameter *N*. Experimental results(Figure 8) showed that the overall accuracy (OA) showed a fluctuating upward trend as *N* increased, but by *N =* 70 this had stabilized at OA = 95.12% with a calculation time of 3.5 minutes. When *N* > 70, the classification accuracy did not improve effectively, while the calculation time increased significantly, leading to decreased of calculation efficiency. Therefore, we chose *N* = 70 to construct the RF classification model.

#### 2.3.4. Accuracy Analysis of Multi-temporal and Multi-feature Tea Plantation Identification Method 

In order to determine the best scheme for tea plantation identification, eight groups of feature models (Table 2) were designed based on multi-temporal and multi-feature characteristics.

We then compared the classification results of the different models (Table 3). Generally speaking, the classification accuracy showed an upward trend with increasing types of feature variables. The producer’s accuracy and the overall accuracy for both tea plantation types in the multi-temporal spectral feature model S increased by 18.01%, 22.08%, and 9.61%, respectively, when compared with the single-temporal spectral feature model (S1–4).With regard to S, S + NDVI + DEM, and S + NDVI + DEM + GLCM, the producer’s accuracies of monoculture tea plantations were 93.47%, 93.91%, 94.85, those for polyculture tea plantations were 81.19%, 82.01%, 82.24%, and the overall accuracies were 95.89%, 96.05% and 96.33%, respectively. The advantages of the multi-source information clearly complemented one other, which was conducive to increasing the separability of different LULC and improving the recognition accuracy of tea plantations and the overall classification effect.

#### 2.3.5. Optimum Recognition Features for Tea Plantations

Although the classification accuracy of the S+NDVI+DEM+GLCM model was highest, its abundant feature variables resulted in a low calculation efficiency, making it necessary to select the optimum recognition features from the full set of 325. There are several methods for finding the optimal feature combination. One such method is the backward feature elimination algorithm [31]. Another method involves ranking the feature importance value and accumulating features one by one to the classifier, then selecting the feature subset with the highest accuracy [32]. Owing to the high feature dimension in this study, in order to improve the computational efficiency, we adopted a threshold segmentation method that considers the feature importance value and the number of bands.

Following RF classification feature importance analysis, we carried out experiments with different numbers of features. Because the RF algorithm was a random selection of samples and features, the results of each calculation were different, so the mean values of 10 calculations were obtained to avoid randomness errors. We then ranked the mean values of feature importance and selected features in four importance classes as input features for RF classification (Table 4).

Using 10 features with average importance over 1.00 resulted in a better performance than any single-temporal spectral feature (Table 3), with an overall classification accuracy of 95.01%. Using 17 features with average importance over 0.90 produced an overall accuracy of 95.68%. Using 28 features with average importance over 0.80, produced results close to those achieved when using all 325 feature classifications. This was due to the addition of optimized multi-temporal spectral, NDVI, textural, and topographic features, which increased the spectral differences and separability between different objects. After feature selection, the redundant information was eliminated, and band information that played a key role in classification was retained; this greatly reduced the dimension of input features and effectively reduced the computational complexity of the classifier while achieving high classification accuracy. 

**Figure 9:**
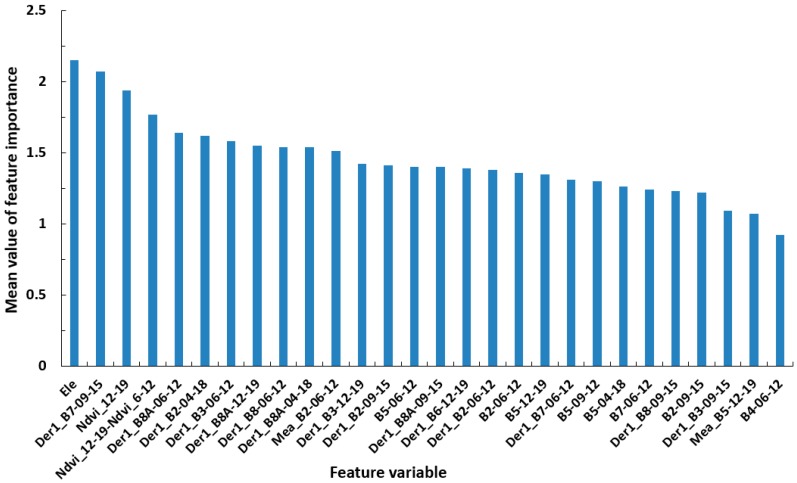
Feature importance ranking for the optimal feature combination.

In order to further explore the impact of each feature variable on classification accuracy, the optimal feature combination was used to identify tea plantations in the study area and calculate the importance of all 28 feature variables, which varied greatly (Figure 9). Ele, Der1_B7-09-15, Ndvi_12-19, and Ndvi_12-19-Ndvi_6-12 had the greatest importance, indicating that elevation, the first derivative of the red-edge band (B7) on September 15, winter NDVI, and the difference between winter and summer NDVI had the greatest contribution to the identification of tea plantations. Seven first spectral derivative and mean texture features (Der1_B8A-06-12, Der1_B2-04-18, Der1_B3-06-12, Der1_B8A-12-19, Der1_B8-06-12, Der1_B8A-04-18, and Mea_B2-06-12) contributed clearly and equally to classification. Overall, there were 8 spectral features, 15 first derivative spectral features, 2 NDVI features, 1 terrain feature, and 1 texture feature; only mean texture contributed to classification and its contribution was low. All four temporal phase features contributed to tea plantation identification, but spectral features in June and September contributed the most.

## 3. Results and Discussion

### 3.1. Classification Results and Accuracy Assessment

**Figure 10:**
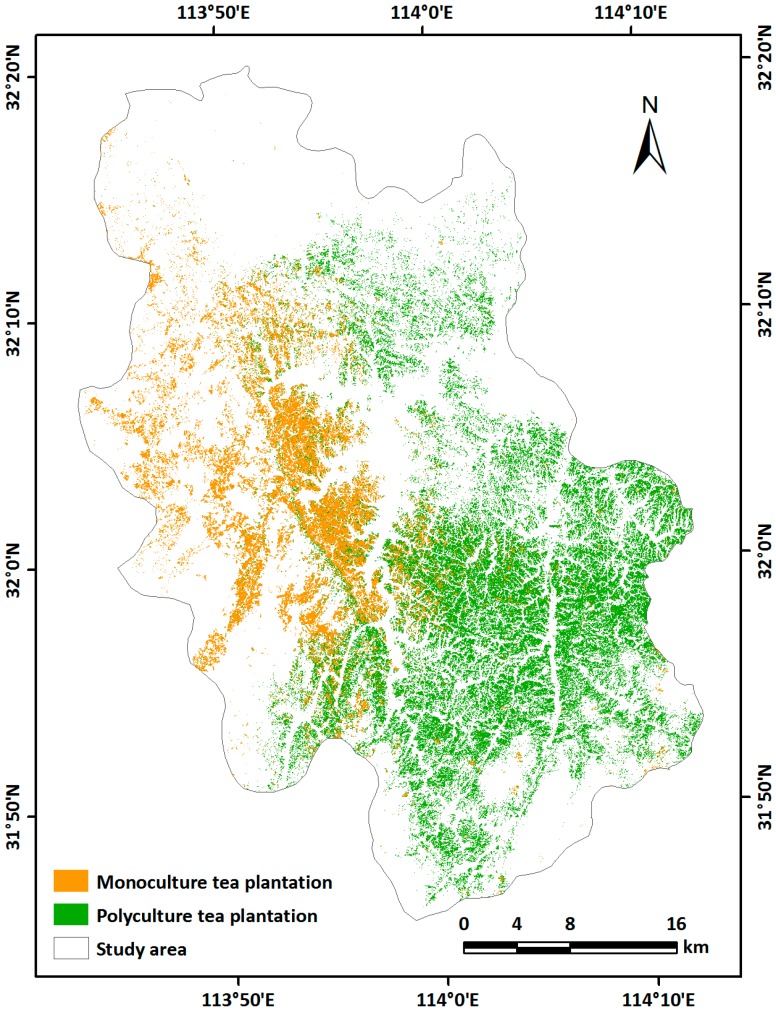
Final map of tea plantations in the study area.

Figure 10 shows the extraction result for tea plantations in the study area using the 28 optimal features with the RF algorithm. In order to assess the overall result, we merged both tea plantation types and analysed the confusion matrix of the classification results. Of the 2449 tea plantation pixels, 2365 were correctly extracted and 84 were misclassified as other LULC types, and 98 of the other 6307 pixels were misclassified as tea plantations. Those misclassified as tea plantations were mainly forest, indicating the serious confusion between these types that affects the accuracy of tea plantation identification; this occurred mainly because of widespread tea plantations interplanting with other agroforestry in the study area. In April, June, and September, the polyculture tea plantations were interplanted with and almost covered by agroforestry. Although these can be distinguished using December imagery, some confusion remained between these very similar types.

### 3.2. Comparison and Analysis Classification Method Accuracy

In order to evaluate the RF method’s performance for tea plantation identification, we used the same data (28 optimal feature combinations designated above) to identify tea plantations using the SVM algorithm and compared the results (Table 5). The overall accuracy of the RF method was 1.49% higher and the producer’s and user’s accuracy for tea plantations were 4.12% and 3.57% higher, respectively, while the classification accuracy of other LULC types was also improved. By comparing the tea plantation area extracted in this paper with that in the 2017 Xinyang Statistical Yearbook [17], we determined that the tea plantation areas extracted by RF and SVM were 44,198 ha and 41,829 ha, respectively, while the statistical area of tea plantations in the study area was 47,256 ha. The relative errors of the RF and SVM methods were 6.47% and 11.48%, respectively, demonstrating the improved performance of the former for tea plantation identification. In addition, the highest accuracy of tea plantation recognition using high-resolution imagery reported in the existing literature reached 95.51%, while that of medium-resolution imagery reached 88.2%; this shows that the RF classification algorithm combined with multi-temporal and multi-feature analysis of medium-resolution images was effective in extracting tea plantation areas.

## 4. Conclusions

We developed a new approach to identifying and classifying tea plantations and tested this using multi-temporal Sentinel-2 remote sensing imagery from the Shihe District of Xinyang City, Henan Province, China. We used the distinct phenological cycles of tea management (multiple annual periods of growth, harvest, and pruning), as well as the distinct characteristics of monoculture and polyculture tea plantations, to extract the initial classification features for eight typical LULC types in the area. These features included spectral reflectance, first derivative spectral features, temporal variations in NDVI, and textural and topographic features. Feature selection was carried out with the RF classification feature importance algorithm, then the RF classifier was used to extract tea plantation areas, with the following conclusions:

(1) The combination of multi-temporal and multi-feature classification methods improved the overall accuracy and tea plantation classification producer’s and user’s accuracies compared with using single-temporal spectral features.
(2) Selecting features using RF importance classification reduced the dimension of input features and the computational complexity, resulting in improved classification efficiency and accuracy. 28 features with average importance >0.80 were selected as optimal features, resulting in an overall classification accuracy of 97.92%, and the producer’s and user’s accuracy for tea plantations of 96.57% and 96.02%, respectively. The classification accuracy was similar to that achieved using 325 initial features before feature selection.
(3) Comparing the classification accuracy of the RF and SVM methods for tea plantation identification, the former’s overall accuracy was 1.49% higher and the producer’s and user’s accuracies were 4.12% and 3.57% higher, respectively.

Further research should focus on two areas. First, both RF and SVM are shallow machine learning algorithms, but the use of deep learning algorithms should be tested for the extraction of tea plantations to further improve recognition accuracy. Second, our methods should be tested and verified in other tea districts and at a larger scope.

## Figures and Tables

**Table 1:** Feature parameters extracted from the four Sentinel-2 images and topographic features.

Feature Type	Feature Phase	Feature Name	Feature Variable	Feature Number
Spectral feature	2018-4-18, 2018-6-12, 2017-9-15, 2017-12-19	Reflectance	B2, B3, B4, B5, B6, B7, B8, B8A	32
		First derivative spectral	Der1_B2, Der1_B3, Der1_B4, Der1_B5, Der1_B6, Der1_B7, Der1_B8, Der1_B8A	32
Vegetation index	2018-6-12, 2017-12-19	NDVI	Ndvi_12-19, Ndvi_12-19- Ndvi_6-12	2
Texture feature (GLCM)	2018-4-18, 2018-6-12, 2017-9-15, 2017-12-19	Mean	Mea_B2, Mea_B3, Mea_B4, Mea_B5, Mea_B6, Mea_B7, Mea_B8, Mea_B8A	32
		Variance	Var_B2, Var_B3, Var_B4, Var_B5, Var_B6, Var_B7, Var_B8, Var_B8A	32
		Contrast	Con_B2, Con_B3, Con_B4, Con_B5, Con_B6, Con_B7, Con_B8, Con_B8A	32
		Homogeneity	Hom_B2, Hom_B3, Hom_B4, Hom_B5, Hom_B6, Hom_B7, Hom_B8, Hom_B8A	32
		Dissimilarity	Dis_B2, Dis_B3, Dis_B4, Dis_B5, Dis_B6, Dis_B7, Dis_B8, Dis_B8A	32
		Correlation	Cor_B2, Cor_B3, Cor_B4, Cor_B5, Cor_B6, Cor_B7, Cor_B8, Cor_B8A	32
		Entropy	Ent_B2, Ent_B3, Ent_B4, Ent_B5, Ent_B6, Ent_B7, Ent_B8, Ent_B8A	32
		Angular second moment	Asm_B2, Asm_B3, Asm_B4, Asm_B5, Asm_B6, Asm_B7, Asm_B8, Asm_B8A	32
Topographic feature	-	Elevation	Ele	1
		Slope	Slo	1
		Aspect	Asp	1
Sum				325

**Table 2:** Eight groups of feature models used for accuracy analysis.

Feature Model	Feature Dimension	Description
S1	16	8-band spectral features on 2018-4-18
S2	16	8-band spectral features on 2018-6-12
S3	16	8-band spectral features on 2017-9-15
S4	16	8-band spectral features on 2017-12-19
S	64	8-band spectral features of all four images
GLCM	256	8-band texture features of all four images
S+NDVI+DEM	69	8-band spectral features of all four images + Vegetation index features + Topographic features
S+NDVI+DEM+GLCM	325	8-band spectral features of all four images + Vegetation index features + Topographic features + 8-band texture features of 4 phases

**Table 3:** Comparison of classification accuracy of different feature models using the RF method.

Feature Model	Monoculture Tea Plantation		Polyculture Tea Plantation		OA/%	Kappa
	PA /%	UA /%	PA /%	UA /%		
S1	75.46	71.76	59.11	61.71	86.28	0.6890
S2	87.13	83.92	69.28	58.83	89.06	0.7585
S3	80.92	83.00	74.77	63.24	88.88	0.7512
S4	85.56	77.71	73.60	70.63	89.60	0.7693
S	93.47	92.71	81.19	84.45	95.89	0.9059
GLCM	89.14	91.55	76.64	74.97	93.38	0.8485
S+NDVI+DEM	93.91	92.92	82.01	83.08	96.05	0.9099
S+NDVI+DEM+GLCM	94.85	93.56	82.24	85.02	96.33	0.9163

**Table 4:** Accuracy comparison of classification results with different numbers of optimum features.

Mean Value of Feature Importance	Feature Dimension	Monoculture Tea Plantation		Polyculture Tea Plantation		OA/%	Kappa
		PA /%	UA /%	PA /%	UA /%		
≥1.00	10	93.28	90.28	78.39	78.76	95.01	0.8870
≥0.90	17	94.10	91.46	80.37	81.71	95.68	0.9020
≥0.80	28	94.29	91.75	81.66	84.62	96.05	0.9100
≥0.75	39	93.28	91.81	81.19	82.94	95.91	0.9068

**Table 5:** Comparison of classification accuracies of the RF and SVM methods.

Classes	RF		SVM	
	PA /%	UA /%	PA /%	UA /%
Tea plantation	96.57	96.02	92.45	92.45
Others	98.45	98.67	97.97	97.09
OA/%	97.92		96.43	
Kappa	0.9485		0.9107	
Feature dimension	28		28	
